# Atraumatic femoral head necrosis: a biomechanical, histological and radiological examination compared to primary hip osteoarthritis

**DOI:** 10.1007/s00402-021-03890-4

**Published:** 2021-05-10

**Authors:** Alexander Hofmann, Benjamin Fischer, Stefan Schleifenbaum, Sascha Kurz, Melanie Edel, Gudrun Borte, Gabriele Lehmann, Andreas Roth

**Affiliations:** 1 Department of Orthopaedic, Trauma and Plastic Surgery, Leipzig University, Liebigstr. 20, 04103 Leipzig, Germany; 2 ZESBO—Center for Research on the Musculoskeletal System, Leipzig University, Semmelweisstraße 14, 04103 Leipzig, Germany; 3 Section Rheumatology/Osteology, Department of Internal Medicine III, University Hospital Jena, Am Klinikum 1, 07740 Jena, Germany

**Keywords:** Femoral head necrosis, Biomechanical evaluation, Histological evaluation, Thin-slice CT, Primary hip osteoarthritis

## Abstract

**Introduction:**

Atraumatic necrosis of the femoral head (AFHN) is a common disease with an incidence of 5000–7000 middle-aged adults in Germany. There is no uniform consensus in the literature regarding the configuration of the bone in AFHN. The clinical picture of our patients varies from very hard bone, especially in idiopathic findings, and rather soft bone in cortisone-induced necrosis. A better understanding of the underlying process could be decisive for establishing a morphology-dependent approach. The aim of this study is the closer examination of the condition of the bone in the AFHN compared to the primary hip osteo arthritis (PHOA).

**Materials and methods:**

The preparations were obtained as part of elective endoprosthetic treatment of the hip joint. Immediately after sample collection, thin-slice CT of the preserved femoral heads was performed to determine the exact density of the bone in the necrosis zone. Reconstruction was done in 0.8–1 mm layers in two directions, coronary and axial, starting from the femoral neck axis. Density of the femoral heads was determined by grey value analysis. The value in Hounsfield units per sample head was averaged from three individual measurements to minimize fluctuations. For biomechanical and histomorphological evaluation, the samples were extracted in the load bearing zone perpendicular to the surface of the femoral head. Group-dependent statistical evaluation was performed using single factor variance analysis (ANOVA).

**Results:**

A total of 41 patients with a mean age of 64.44 years were included. The mean bone density of the AFHN samples, at 1.432 g/cm^3^, was about 7% higher than in the PHOA group with a mean value of 1.350 g/cm^3^ (*p* = 0.040). The biomechanical testing in the AFHN group showed a 22% higher—but not significant—mean compressive strength (20.397 MPa) than in the PHOA group (16.733 MPa). On the basis of histological analysis, no differentiation between AFHN and PHOA samples was possible.

**Conclusions:**

The present study (NCT, evidence level II) shows that AFHN has a very well detectable higher bone density compared to PHOA. However, neither biomechanical stress tests nor histomorphological evaluation did show any significant difference between the groups. The results allow the conclusion that there is no “soft” necrosis at all in the AFHN group.

## Introduction and background

With an incidence of 5000–7000 middle-aged adults in Germany, atraumatic necrosis of the femoral head (AFHN) is a common disease with often unclear etiology [14]. Risk factors include cortisone therapy, alcohol abuse, chemotherapy and immunosuppression, for example, after kidney transplantation [14]. Even rarer disease patterns, such as sickle cell anaemia, Gaucher’s disease, lupus erythematosus, or chronic pancreatitis, seem to be associated with an increased incidence of AFHN [1]. Early diagnosis is crucial for a joint-preserving, conservative therapy.

A stadium classification was first made by Ficat and Arlet in the 1960s [6]. The currently clinically applied ARCO classification was first published in 1991 by the Association Research Circulation Osseous [8].

There is no uniform consensus in the literature on the configuration of the bone in advanced femoral head necrosis. The cascade of development of the AFHN begins with an accumulation of fat and displacement of the bone marrow. This is followed by a compression of the venous outflow with a stasis of the venous blood flow, which finally results in ischemia [16]. The early stages of AFHN are clinically and radiologically asymptomatic [16]. Investigations on the pathogenesis of AFHN showed an increased bone-marrow pressure in the radiologically inconspicuous stages and assume a kind of osseous compartment syndrome due to the shown ischemia and perishing of the bone trabecula [10].

## Research question

Previous studies assume a soft form of bone necrosis in AFHN [4], whereas no significant biomechanical differences to healthy bone have been shown in primary hip osteoarthritis (PHOA) [3]. Our own clinical experience, on the other hand, shows only very hard bone, especially in idiopathic findings, and rather soft bone in cortisone-induced necrosis. However, the treatment algorithm of the AFHN is described in the literature in an essentially uniform manner, independent of the local structure and independent of the aetiology. Differences in morphology, histology, and biomechanical properties between AFHN and PHOA would be the basis for explaining the different effectiveness of different procedures and could be decisive for establishing a morphology-dependent approach.

Therefore, the aim of the study is first of all the closer examination of the condition of the bone in AFHN compared to PHOA. Biomechanically, both groups will be examined for differences in the maximum load to failure and Young’s modulus. A thin-slice computer tomography (CT) examination will be performed to show possible differences in the density of the material using Hounsfield units (HU). Finally, the specimens will be histologically processed to substantiate the morphological differences to the PHOA.

## Methods

At first, storage and fixation techniques of the preparations were tested in a pre-series. Subsequently, patients were recruited according to the previously defined inclusion and exclusion criteria. Approval was obtained from the ethics committee of the Medical Faculty of Leipzig University. This study was performed in line with the principles of the Declaration of Helsinki. With the patient’s consent, the corresponding preparations (AFHN/PHOA) were obtained as part of elective endoprosthetic treatment of the hip joint. At the same time, anamnesis and radiological findings were reviewed and the values for the corresponding variables were entered into a database.

**Fig. 1:**
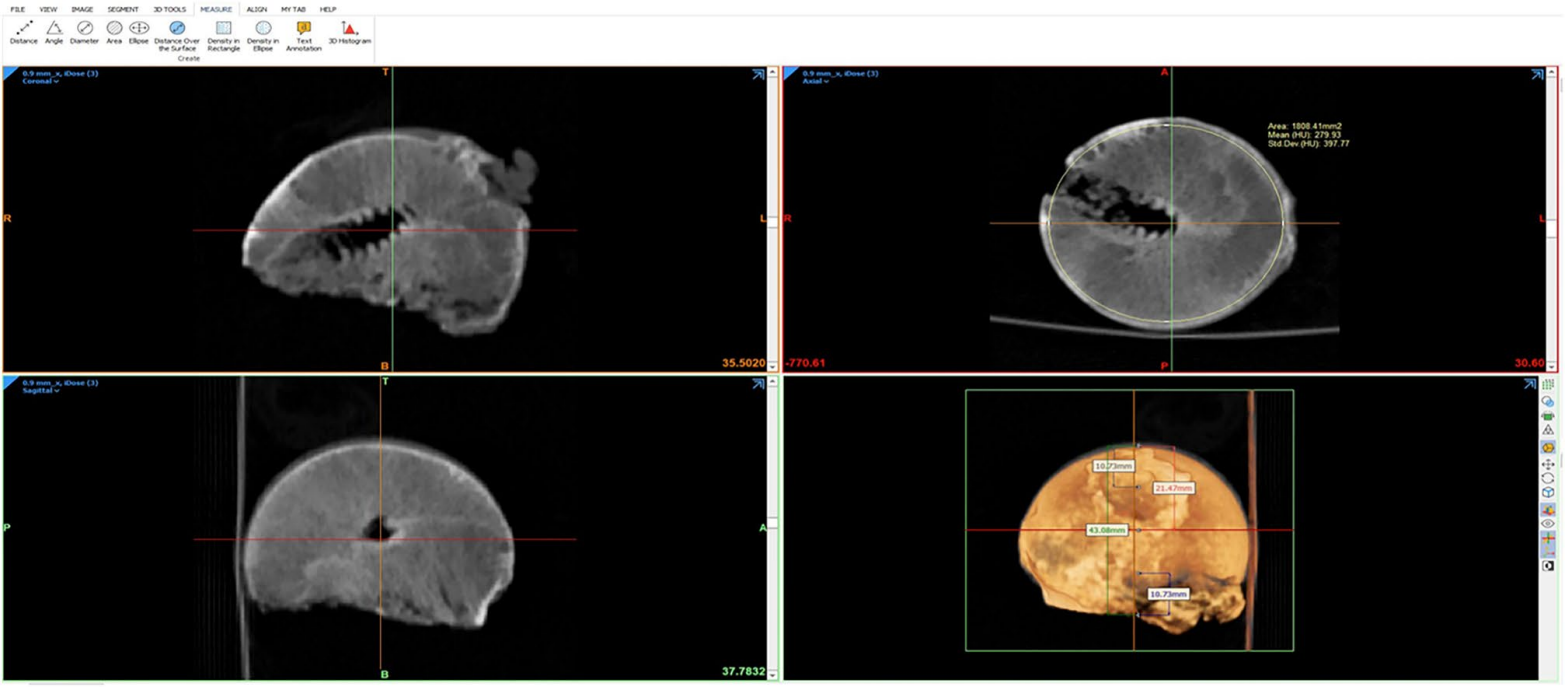
Reconstruction of CT-scans

Immediately following sample collection, thin-slice CT of the preserved femoral heads was performed to determine the exact density of the bone in the necrosis zone. Since solely the tissue samples were examined, the parameters (acceleration voltage, current) could be adjusted for optimal imaging without concern over radiation. The investigation was performed using a Philips iCT 256 (Philips Healthcare) at 120 kV and 50 mAs. Reconstruction was done in 0.8–1 mm layers in two directions, coronary and axial, starting from the femoral neck axis (Fig. 1). Density of the femoral heads was determined by grey value analysis (Materialise Mimics, Materialise NV, Leuven, Belgium). The value in Hounsfield units per sample head was averaged from three individual measurements to minimize fluctuations.

Afterwards, the samples were fixed in acetone and stored. For biomechanical testing, the samples were extracted using a hollow drill in the load bearing zone perpendicular to the surface of the femoral head, cut to a length of 10 mm using a band saw (EXAKT 310, EXAKT Advanced Technologies GmbH, Norderstedt, Germany) and stored in acetone for 24 h at room temperature. Due to cramped geometric conditions and destruction of the surrounding bone area during the drilling process the collection of several drill cores per femoral head was not possible. A cylindrical sample size was chosen due to the simplicity of handling using the non-cooled hollow drill. The specimen length of 10 mm was chosen, because, with a height–width ratio of 1:1, it provided a good compromise between usable material characteristics, stability during testing after initial trials and geometric aspects [3, 17]. Specimens were tested using a uniaxial compression testing machine (Instron 5566A, Instron Corporation, Norwood, MA, USA). For testing, a 2.5 kN load cell and a path-controlled test method with a fixed test speed of 0.1 mm/min/mm of specimen height, i.e., 1 mm/min was chosen (Bluehill 2 testing software, Instron Corporation, Norwood, MA, USA). The necrotic state of the tissue did not allow a reliable prediction of the mechanical behaviour—therefore, no preconditioning was performed with sample compression excessing 40% testing ended automatically. Finally, the group-dependent statistical evaluation of the results was performed in the form of descriptive statistics and a comparison of the mean values using analysis of variance (ANOVA).

The samples for the histomorphological examination were embedded in methyl methacrylate [2] and cut in 5 µm slices using a microtome (Reichert-Jung Polycut S, Nussloch, Germany). To differentiate between mineralized and non-mineralized bone, the modified Masson–Goldner Trichrome staining was used [9]. Microscopic evaluation was performed according to the previously determined parameters bone volume (BV/TV—% of bone volume in total tissue), trabecular structure, detection of non-mineralized bone (osteoid) on the endosseous surface, presence of osteoblasts and osteoclasts, active osteoclasis, empty reserve lacunae, and fibrosis. Findings were documented in table form.

## Results

Forty-one patients with a mean age of 64.44 years were included (24 male, 17 female). In 19 cases, the right hip joint was affected, and in 22 cases the left hip joint. Among others, the following secondary diseases were found in descending order of frequency: arterial hypertension, renal insufficiency, cortisone therapy, renal insufficiency, chemotherapy, and steatosis hepatis. All patients agreed in written consent to participate in the study.

A total of 38 samples (14 AFHN, 24 PHOA) could be obtained for CT-analysis, 20 samples (16 PHOA, four AFHN) for biomechanical testing and 19 (AFHN five, PHOA 14) for histological examination. Further drill cores could not be collected without defects and were, therefore, not available for analysis.

Thin-slice computer tomography results (mean in Hounsfield units) as well as the calculated bone density are shown in Table 1.

**Table 1:** Thin-film CT group-dependent chart of the mean values with SD and significance levels

Sample type	*n*	Mean value	Standard deviation	*p*
Hounsfield units				
AFHN	14	437.067	155.013	0.040
PHOA	24	355.011	83.641	
Total	38	385.242	120.002	
Density [g/cm^3^]				
AFHN	14	1.432	0.154	0.040
PHOA	24	1.350	0.083	
Total	38	1.380	0.119	

**Fig. 2:**
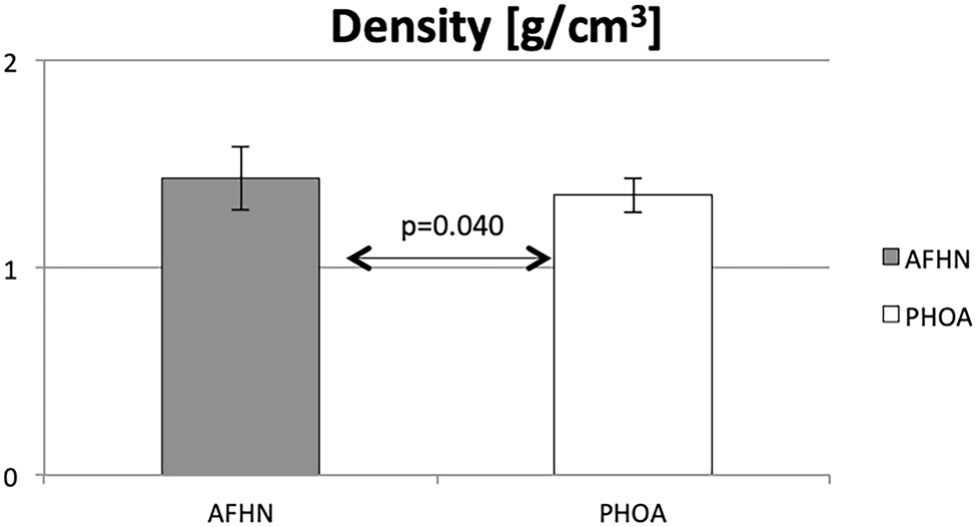
Bone density—group-dependent plots of the mean values with SD (*p* = 0.040)

The mean bone density of the AFHN samples, at 1.432 g/cm^3^, was about 7% higher than in the PHOA group with a mean of 1.350 g/cm^3^ (*p* = 0.040, Fig. 2).

The results of biomechanical testing (mean and SD for compressive strength, failure compression, and Young’s modulus) are shown in Table 2. During one measurement, the maximum of the load cell was reached, and this measurement was rejected subsequently.

**Table 2:** Biomechanical testing: group-dependent chart of the mean values with SD and significance levels

Sample type	*n*	Mean value	Standard deviation	*p*
Compressive strength [MPa]				
AFHN	3	20.397	18.518	0.587
PHOA	16	16.733	8.918	
Total	19	17.312	10.308	
Young’s modulus [MPa]				
AFHN	3	8.429	3.875	0.531
PHOA	16	7.342	2.503	
Total	19	7.514	2.656	
Failure compression [%]				
AFHN	3	4.414	0.477	0.208
PHOA	16	3.673	0.943	
Total	19	3.790	0.919	

**Fig. 3:**
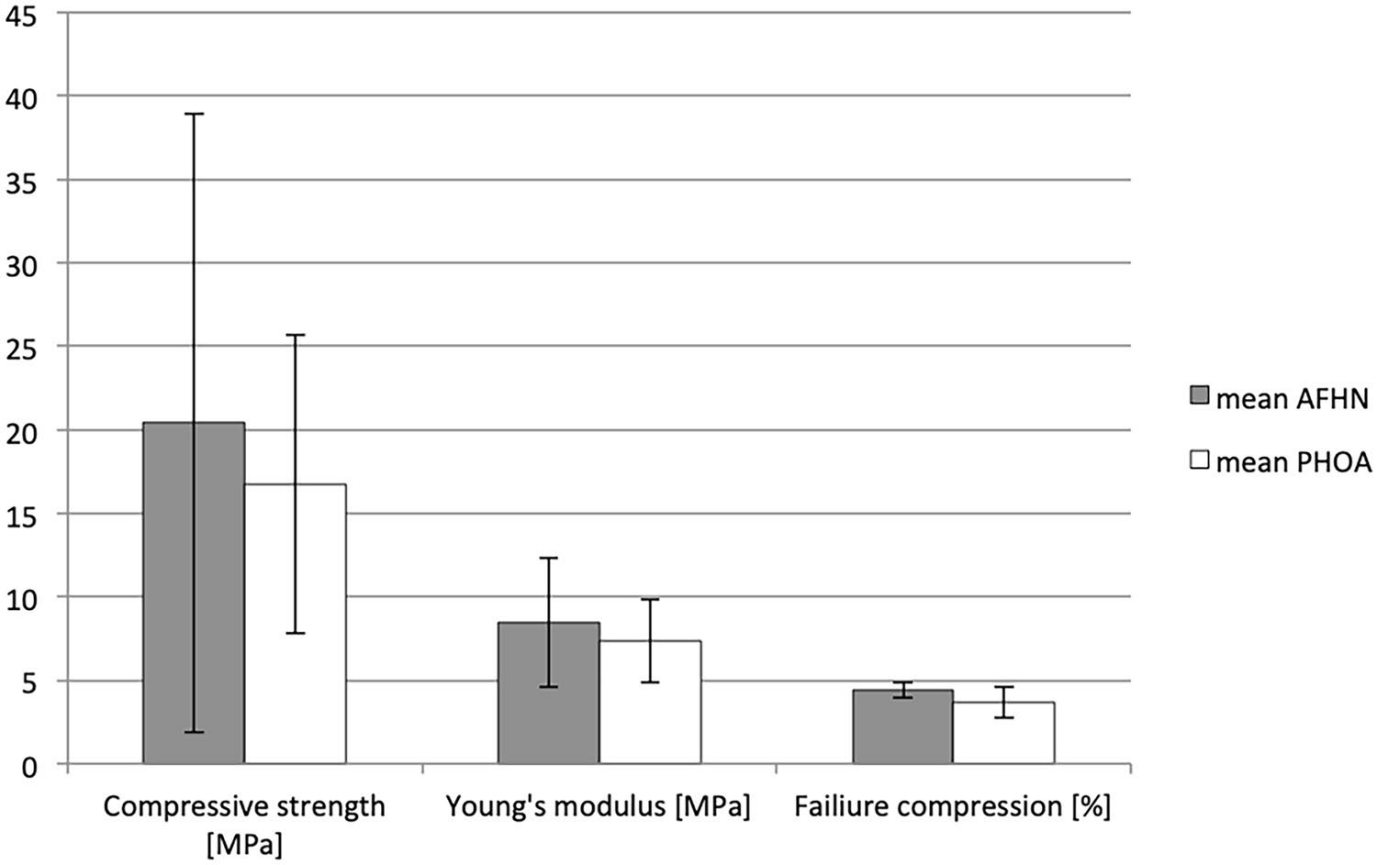
Biomechanical testing—group-dependent plots of mean values with SD

The mean compressive strength of the AFHN samples was 20.397 MPa, about 21.9% higher than in the PHOA group (mean 16.733 MPa). The mean modulus of elasticity was 8.429 MPa in the AFHN group and 7.342 MPa in the PHOA group, and the failure compression was 4.414% in the AFHN group and 3.673% in the PHOA group. However, no statistically significant differences could be shown (Fig. 3).

Histomorphological examination showed a normal or decreased proportion of bone volume to total sample volume (BV/TV) in almost equal parts in PHOA and AFHN samples; increased bone volume was observed in only one sample (PHOA) (Table 3). The trabecular network was destroyed in nine PHOA samples. It was preserved in five PHOA samples and in three of the AFHN samples. The detection of osteoid on the endosteal surface was successful in four of the 14 PHOA samples and in two of the four AFHN samples. A similar distribution was found for the detection of osteoblasts and osteoclasts at the endosteal surface. The samples with active bone resorption showed different degrees of endosteal fibrosis. With the exception of two PHOA samples, all samples showed empty resorption lacunea as an indication of former osteoclastic degradation (Table 3). Histologically, no significant differentiation between AFHN and PHOA samples could be observed.

**Table 3:** Results of the histomorphological examination

No.	Type	BV/TV	Trab structure	OS/ BS	Osteo-blasts	Osteo-clasts	Active osteo-clasis	Empty resection lacunae	Fibrosis
1	PHOA	Normal	Destroyed	Yes	Yes	Yes	Yes	Yes	No
2	PHOA	Normal	Preserved	No	Yes	Yes	Yes	Yes	Yes
3	AFHN	Normal	Preserved	No	No	Yes	Yes	Yes	Yes
4	AFHN	Only cortical bone		Yes	Yes	No	No	Yes	Slightly
5	PHOA	Normal	Destroyed	1 area	Yes	No	No	Yes	No
6	AFHN	Normal	Preserved	No	No	Yes	Yes	Yes	Slightly
7	PHOA	Normal	Preserved	No	No	No	No	Yes	No
8	PHOA	Normal	Intact	No	No	Yes	Yes	Yes	Yes
9	PHOA	Increased	Preserved	No	No	No	No	Yes	Slightly
10	PHOA	Reduced	Destroyed	No	No	No	No	Yes	No
11	PHOA	Reduced	Destroyed	No	No	No	No	Yes	No
12	PHOA	Reduced	Destroyed	Yes	Yes	Yes	Yes	Yes	No
13	AFHN	Reduced	Destroyed	Yes	Yes	Yes	Yes	Yes	Yes
14	PHOA	Reduced	Destroyed	No	No	No	No	No	No
15	AFHN	Normal	Intact	Yes	No	No	No	Yes	No
16	PHOA	Reduced	Destroyed	No	No	No	No	No	No
17	PHOA	Reduced	Destroyed	No	No	No	No	Yes	Slightly
18	PHOA	Normal	Destroyed	No	No	No	No	Yes	No
19	PHOA	Normal	Preserved	No	No	No	No	Yes	No

## Summary of the results

CT scans showed a significantly higher mean bone density of the AFHN samples than in the PHOA group (1.432 g/cm^3^ vs 1.350 g/cm^3^, *p* = 0.040). The biomechanical testing in the AFHN group showed a 22% higher—but not significant—mean compressive strength (20.397 MPa) than in the PHOA group (16.733 MPa). On the basis of histological analysis, no differentiation between AFHN and PHOA samples was possible.

## Discussion

The present study is a non-randomized, controlled study (NCT, evidence level II). Examination of the tissue samples using thin-layer CT showed statistically significant differences in the mean bone density of the femoral head between PHOA and AFHN groups calculated from Hounsfield units. Due to mineral enrichment as a result of the progressive calcification of the necrotic bone marrow, there is a radiologically detectable bone densification in the area of necrosis [15].

To support these data, increased compressive strength and increased force to failure would have been expected in the biomechanical investigation. However, the compressive strengths determined in the PHOA group showed considerable inter-individual differences. Even in femoral head samples with existing AFHN, considerable interindividual differences were found. Surprisingly, statistical differences between the two groups could not be shown.

The results are, therefore, not reliable enough for a comprehensive statement regarding the compressive strength or hardness of the AFHN. Even the histological evaluation could not support our hypothesis. Especially regarding the different literature statements on the nature of the AFHN, a further investigation on a larger number of samples is necessary to get valid evidence.

These results lead to several possible conclusions. On one hand, it must be clarified in the future whether there may be different morphological types of AFHN depending on etiology. It cannot be ruled out that depending on the etiology necrosis, which is accompanied by a granulation tissue and is soft, may also occur. Furthermore, the fact that we have found that hard bone tissue allows the question whether in case of core decompression large diameters for drilling have to be chosen in principle (and be filled with autologous spongiosa or bone substitutes) to achieve a better regeneration than with the use of drills with small diameters.

Looking at the literature, a histological comparison of the AFHN with the much more frequently occurring PHOA showed similarities and differences: hypoxia of the subchondral bone and signs of necrosis of cancellous bone and bone trabeculae could be shown in both clinical pictures [13]. Intraosseous thrombosis also occurs in both diseases [5]. However, a difference was found regarding the intraosseous pressure. For PHOA, normal pressure levels were observed, whereas the intraosseous pressure was significantly increased in the AFHN [13]. However, the carbon dioxide partial pressure proved to be normal in both cases [13].

A biomechanical investigation of the AFHN by TD Brown et al. [4] showed a reduced maximum force and a significantly reduced Young’s modulus with slightly increased maximum load to failure in comparison to healthy femoral head samples. Another examination showed decreasing values for Young’s modulus and the maximum load to failure with increasing AFHN stage [11]. Investigations on the biomechanical characteristics of PHOA showed no significant differences with regard to Young’s modulus and maximum load to failure in comparison to healthy bone [3].

The histological evaluation allowed no differentiation between PHOA and AFHN samples. This could be due to the numerous concomitant diseases affecting bone metabolism. The examined parameters only allow a limited conclusion on the metabolic rate of the bone—a more precise statement on bone metabolism would be possible after marking with tetracycline prior to the examination. In addition, standard values for static and dynamic histological parameters exist so far only for bone extracted from the iliac crest.

A key limitation of our examinations is the extraction technique of the femoral head by the surgeon, as it became clear in the CT examinations. The instruments used here cause a more or less large spongy substance defect. This has a decisive influence on the number of cores available later, as these are to be removed exclusively from intact cancellous bone. For further investigations, we suggest a secondary osteotomy after dislocation of the femoral head without using an extractor instrument to keep the bone structure intact.

A limitation of the biomechanical examination is that even small deviations of the drilling angle from the main load direction in the femoral head can lead to large interindividual differences in bone characteristics, since the structure and strength of the cancellous bone are subject to a strong anisotropy [12]. The fixation technique of the bone also has an influence on the structure of the bone and subsequently the measurement results [7]. Not least, regarding to the small number of samples and the associated large standard deviations, no significant differences could be shown in the biomechanical tests. This applies also to the histological evaluation, where a significantly larger number of samples would be required to make a distinct statement regarding an etiologic differentiation.

In summary, the present study could show that the AFHN has a significant higher bone density compared to PHOA, very well detectable by computer tomography. The biomechanical stress tests, however, did not show significant differences between the groups. For the increased bone density in the AFHN group, no histological correlation could be found. The results allow the conclusion that AFHN does not come with a “soft” form of necrosis typically. However, the hypothesis that this results in a significantly higher maximum load to failure in the biomechanical examination had to be rejected.

## Abbreviations

AFHN: Atraumatic femoral head necrosis
PHOA: Primary hip osteoarthritis
CT: Computer tomography
Mpa: Megapascal
OS: Osteoid surface area
BS: Bone surface area
BV/TV: % Of bone volume in tissue
