# Correction to: Atraumatic femoral head necrosis: a biomechanical, histological and radiological examination compared to primary hip osteoarthritis

**DOI:** 10.1007/s00402-021-03995-w

**Published:** 2021-06-24

**Authors:** Alexander Hofmann, Benjamin Fischer, Stefan Schleifenbaum, Sascha Kurz, Melanie Edel, Gudrun Borte, Gabriele Lehmann, Andreas Roth

**Affiliations:** 1 Department of Orthopaedic, Trauma and Plastic Surgery, Leipzig University, Liebigstr. 20, 04103 Leipzig, Germany; 2 ZESBO—Center for Research on the Musculoskeletal System, Leipzig University, Semmelweisstraße 14, 04103 Leipzig, Germany; 3 Section Rheumatology/Osteology, Department of Internal Medicine III, University Hospital Jena, Am Klinikum 1, 07740 Jena, Germany

## Correction to: Archives of Orthopaedic and Trauma Surgery 10.1007/s00402-021-03890-4

The original version of this article unfortunately contained a mistake. The given name and family name of authors were swapped. The correct given name and family name should be:

Alexander Hofmann · Benjamin Fischer · Stefan Schleifenbaum · Sascha Kurz · Melanie Edel · Gudrun Borte · Gabriele Lehmann · Andreas Roth

The original article has been corrected.

